# Sensory Difficulties in Children With an FMR1 Premutation

**DOI:** 10.3389/fgene.2018.00351

**Published:** 2018-08-28

**Authors:** Melissa Raspa, Amanda Wylie, Anne C. Wheeler, Jacek Kolacz, Anne Edwards, Keri Heilman, Stephen W. Porges

**Affiliations:** ^1^RTI International, Durham, NC, United States; ^2^Traumatic Stress Research Consortium, Kinsey Institute, Indiana University Bloomington, Bloomington, IN, United States; ^3^Department of Psychiatry, The University of North Carolina at Chapel Hill, Chapel Hill, NC, United States

**Keywords:** FMR1 premutation, fragile X syndrome, sensory processing, hypersensitivity, hyposensitivity, feeding behavior

## Abstract

Abnormal sensory processing is one of the core characteristics of the fragile X phenotype. Studies of young children with fragile X syndrome (FXS) and the FMR1 premutation have shown sensory challenges as early as infancy and into early childhood. This study sought to examine differences in sensory difficulties in children with an FMR1 premutation compared with children with FXS and typically developing children. We conducted an online survey of 176 parents of affected children (FXS or FMR1 premutation). Most respondents were mothers who are Caucasian (86%), have a 4-year college or graduate degree (68%), and are married (92%). Children ranged in age from 5 to 18, with a mean age of 13.0 years (3.3 SD). Participants completed the BBC Sensory Scales, a 50-item Likert-type scale (1 = Almost Always, 4 = Almost Never) comprised of 8 subscales that assessed auditory processing, visual processing, tactile processing, and eating and feeding behaviors. Mean scores were calculated for the items and each of the subscales. Non-parametric tests examined differences in child and family-level variables. Across all BBCSS subscales, children with an FMR1 premutation displayed more sensory challenges than typically developing children. For six out of the eight subscales, children with the full mutation had the lowest scores indicating more sensory challenges, but this was closely followed by children with an FMR1 premutation. Fragile X status was associated with seven of the eight subscales; children with an FMR1 premutation did not differ from children with FXS on any of the subscales but had more digestive problems than children with no fragile X. Gender, autism status, and family income were also related to sensory sensitivities. In conclusion, these data provide further evidence that some children with an FMR1 premutation experience sensory difficulties that are similar to children with FXS but different than typically developing children.

## Introduction

Fragile syndrome (FXS) is an inherited condition found in approximately 1 in 4,000 males and 1 in 8,000 females (Riley et al., 2017). FXS results from a mutation on the 5′ untranslated region of the *FMR1* gene, which causes more than 200 repeats of the trinucleotide cysteine-guanine-guanine (CGG). The expansion diminishes the production of fragile X mental retardation protein (FMRP), which is essential for normal brain development. Lack of FMRP leads to intellectual disability and a variety of co-occurring conditions, such as anxiety, attention problems, autism, and other associated medical problems, including seizures and otitis media (Bailey et al., 2008; Kidd et al., 2014). One of the hallmark features frequently described for individuals with FXS is hyperarousal, especially in response to sensory stimuli (Cohen, 1995; Miller et al., 1999). Longitudinal studies have shown that sensory processing problems begin early in life for children with FXS and continue to be a challenge as they get older (Baranek et al., 2008). When compared with typically developing controls, children with FXS often show higher rates of tactile sensitivity, taste/smell sensitivity, stimulation seeking behaviors, and auditory filtering (Rogers et al., 2003). Other common sensory challenges found in children with FXS are selective eating (Raspa et al., 2010) and gastrointestinal issues (Kidd et al., 2014). Sensory issues reported in FXS are statistically similar to children with non-syndromic autism spectrum disorder (McIntosh et al., 1999; Rogers et al., 2003), which may be due to similar pathophysiological and anatomical abnormalities (Belmonte and Bourgeron, 2006; Hagerman, 2006).

Individuals with 55–200 CGG repeats have the *FMR1* premutation, which can expand to the full mutation of FXS in future generations. In addition to reproductive risk, individuals with an *FMR1* premutation may develop two known associated conditions, fragile X-associated tremor/ataxia syndrome and fragile X-associated premature ovarian insufficiency. A growing body of evidence also suggests that some individuals with an *FMR1* premutation may have a similar, although attenuated, phenotype to individuals with the full mutation (Boyle and Kaufmann, 2010; Gallagher and Hallahan, 2012; Wheeler et al., 2017). In an early report, a subset of males with an *FMR1* premutation were found to have developmental, behavioral, and physical characteristics which were similar to those seen in FXS (Aziz et al., 2003). Subsequent studies have found higher rates of autism spectrum disorder or autism symptoms in males with an *FMR1* premutation when compared to both typically developing sibling and non-sibling controls (Farzin et al., 2006; Loesch et al., 2007; Bailey et al., 2008; Chonchaiya et al., 2012). The increased prevalence of autism symptomology in the *FMR1* premutation may be due to mRNA toxicity (Goodlin-Jones et al., 2004; Hagerman et al., 2011). Measures of social cognition, such as facial expression recognition, in individuals with an *FMR1* premutation have also been shown to be worse when compared to non-familial controls (Cornish et al., 2005). Other behavioral challenges, including ADHD (Farzin et al., 2006), obsessive-compulsive symptoms (Hessl et al., 2005), and mood and anxiety disorders (Bourgeois et al., 2011) have been reported at higher rates in individuals with the *FMR1* premutation as well.

Given the similarity across the fragile X spectrum in phenotypic expression with regard to clinical features such as autism and other behavioral issues, increased sensory dysregulation may also be present in individuals with an *FMR1* premutation. Few studies, however, have focused specifically on sensory challenges. One exception is a recent longitudinal study of 26 infants with an *FMR1* premutation identified through newborn screening which found statistically significant differences on hypo- and hyper-responsivity when compared with typically developing controls (Wheeler et al., 2016). This pattern was noted to be more similar to that of infants with FXS and autism than to typically developing infants. Given these initial findings, we wanted to further understand the sensory challenges experienced by children with an *FMR1* premutation using a large national survey sample. The goals of this paper were to: (a) report on sensory processing in children with an *FMR1* premutation as well as those with FXS and typically developing children using a newly developed tool, the Brain Body Center Sensory Scales, and (b) examine differences in sensory processing across a variety of child- and family level variables, including fragile X status, gender, age, autism status, and income level.

## Materials and Methods

### Participants and Recruitment

Families were recruited through an online research registry, *Our Fragile X World*, housed at RTI International. Families were eligible for the survey if they had a child who had the full mutation or premutation. A total of 912 families were invited to participate and 347 parents completed the online survey (38%) on 379 children. Although the survey was open to families with children of all ages, our research questions were focused on children. We therefore limited the analytic sample to children that were younger than 18 years old (*N* = 176). Most respondents were Caucasian (86%), had a 4-year college or graduate degree (68%), had incomes over $75,000 (58%), and were married (92%). Children ranged in age from 5 to 18 with a mean age of 13.0 years (*SD* = 3.3 SD); 70% were male. See **Table 1** for more information about the children in the sample.

**Table 1:** Descriptive information about children in the sample.

	Total sample (*n* = 176) *n* (%)	Typically developing (*n* = 33) *n* (%)	*FMR1* premutation (*n* = 14) *n* (%)	Full mutation (*n* = 129) *n* (%)
Age: mean (*SD*)	13.0 (3.3)	12.7 (3.3)	11.7 (3.2)	13.2 (3.3)
Gender: male	123 (69.9)	17 (51.5)	7 (50.0)	99 (76.7)
Total co-occurring conditions: mean (*SD*)	3.3 (2.3)	0.7 (1.1)	1.6 (2.1)	4.1 (1.9)
**Overall health**				
Excellent	89 (50.6)	26 (78.8)	7 (50.0)	56 (43.4)
Very good	59 (33.5)	6 (18.2)	6 (42.9)	47 (36.4)
Good	20 (11.4)	0 (0.0)	1 (7.1)	19 (14.7)
Fair	8 (4.6)	1 (3.0)	0 (0.0)	7 (5.4)
**Quality of life**				
Excellent	65 (36.9)	20 (60.6)	6 (42.9)	39 (30.2)
Very good	82 (46.6)	10 (30.3)	7 (50.0)	65 (50.4)
Good	25 (14.2)	3 (9.1)	1 (7.1)	21 (16.3)
Fair	4 (2.3)	0 (0.0)	0 (0.0)	4 (3.1)
**Ability to listen and pay attention**				
Very good	37 (21.0)	20 (60.6)	7 (50.0)	10 (7.8)
Good	63 (35.8)	10 (30.3)	4 (28.6)	49 (38.0)
Fair	56 (31.8)	3 (9.1)	3 (21.4)	50 (38.8)
Poor	20 (11.4)	0 (0.0)	0 (0.0)	20 (15.5)
**Ability to act appropriately with others same age**				
Very good	38 (21.6)	25 (75.8)	7 (50.0)	6 (4.7)
Good	24 (13.6)	6 (18.2)	3 (21.4)	15 (11.6)
Fair	54 (30.7)	2 (6.1)	3 (21.4)	49 (38.0)
Poor	60 (34.1)	0 (0.0)	1 (7.1)	59 (45.7)
**Ability to adapt to new situations**				
Very good	30 (17.1)	21 (63.6)	3 (21.4)	6 (4.7)
Good	50 (28.4)	10 (30.3)	5 (35.7)	35 (27.1)
Fair	64 (36.4)	2 (6.1)	4 (28.6)	58 (45.0)
Poor	32 (18.2)	0 (0.0)	2 (14.3)	30 (23.3)
**Overall thinking, reasoning, and learning ability**				
Very good	38 (21.6)	24 (73.7)	8 (57.1)	6 (4.7)
Good	46 (26.1)	7 (21.2)	4 (28.6)	35 (27.1)
Fair	63 (35.8)	2 (6.1)	2 (14.3)	59 (45.7)
Poor	29 (16.5)	0 (0.0)	0 (0.0)	29 (22.5)

### Instruments and Procedures

Upon enrollment into the research registry, respondents were asked to provide basic information about themselves and their children to determine eligibility for future studies. Respondents were asked to update most of this information at the beginning of the current survey. Questions for the respondent included their race and ethnicity, age, income, education, and marital status. For their children, respondents provided information on his/her age, fragile X status (full mutation, premutation, no fragile X, not tested), overall health (1 = *Excellent*, 2 = *Very good*, 3 = *Good*, 4 = *Fair*, 5 = *Poor*), quality of life (1 = *Excellent*, 2 = *Very good*, 3 = *Good*, 4 = *Fair*, 5 = *Poor*), and whether he/she had been diagnosed or treated for a variety of co-occurring conditions (anxiety, attention problems, hyperactivity, aggressiveness, self-injurious behavior, autism, seizures, and depression). Respondents also rated their child’s ability to listen and pay attention to others; act appropriately with others his/her own age; adapt to new situations; and overall thinking, reasoning, and learning ability (1 = *Very good*, 2 = G*ood*, 3 = *Fair*, 4 = *Poor*). Given that this was an online survey, all data are based on parent-report.

For each eligible family that was invited to participate, we preselected two children in the family for whom they would complete the survey on. For the first child, the order of preference for the algorithm was: (a) premutation male under 18 years of age, (b) premutation female under 18, (c) full mutation female under 18, and (d) full mutation male under 18. Parents were asked to complete the survey on a second child as well, with preference given to premutation females under 18, then male and female siblings under 18 with no fragile X. We used this algorithm because the registry sample had more children who were males and those with the full mutation; thus, preference was given to children in the family who had an *FMR1* premutation and/or were female.

Respondents completed the BBC Sensory Scales (BBCSS; Porges, 2012), a 50-item Likert-type scale (1 = *Almost always*, 2 = *Frequently/often*, 3 = *Sometimes/occasionally*, 4 = *Almost never*), to assess auditory, visual, and tactile processing, as well as eating and feeding behaviors sensory challenges for each selected child. The scale was developed using evolutionary neurophysiological organizing principles outlined in the Polyvagal Theory (Porges, 2001, 2007), informed by the physiological atypicality observed in children with FXS (Belser and Sudhalter, 1995; Cohen, 1995; Miller et al., 1999; Roberts et al., 2001; Heilman et al., 2011; Klusek et al., 2015). The items are organized into eight subscales: (a) Auditory threat hypersensitivity, (b) Auditory hyposensitivity to voices, (c) Visual hypersensitivity, (d) Tactile hypersensitivity, (e) Affiliate touch aversion, (f) Selective eating, (g) Ingestive problems, (h) Digestive problems (Kolacz et al., 2018).

### Ethics Approval

Prior to enrollment in the research registry and the beginning of the survey, respondents were given an online consent form. After reading the form, respondents were asked to agree to participate before proceeding to the survey questions. This study was carried out in accordance with the recommendations of the United States Department of Health and Human Services (DHHS) Office for Human Research Protections. The protocol was approved by the RTI International’s Institutional Review Board. All participants gave written informed consent in accordance with the Declaration of Helsinki.

### Data Analysis

Three main sets of analyses were conducted using SAS Enterprise Guide v7.15 (Cary, NC, United States). First, means scores were calculated in the observed sample for each item by fragile X status: *FMR1* premutation (*N* = 14), full mutation (*N* = 129), and no fragile X (*N* = 33). Subscale scores were created by taking the mean of the related items. Medians and interquartile ranges are also reported for the subscales. Next, given the small sample sizes and skewness of the data, we utilized non-parametric tests to examine differences in a variety of child- and family level variables. The Kruskal–Wallis test was used as an omnibus test to determine if there were differences in rank-order sums of the sensory subscales by child’s gender (male vs. female), child’s age (≤12 years old vs. >12 years old), autism status (no autism vs. autism), and family income (<$75,000 vs. ≥$75,000). We then used the Kruskal–Wallis omnibus test to examine for possible rank-order sum differences on the BBCSS subscales by fragile x-status (no fragile X, *FMR1* premutation, or full mutation). Omnibus tests that showed a significant difference by fragile X status were followed by a series of three *post hoc* Kruskal–Wallis tests, each comparing two of the groups. We employed the Bonferroni correction at this level of analysis to reduce the increased risk of type 1 error associated with conducting multiple tests.

## Results

### Description of Sensory Behaviors

**Table 2** reports observed mean subscale and item-level scores on the BBCSS by fragile X status. Mean subscale scores for children with an *FMR1* premutation ranged from 2.95 to 3.67. Children with the full mutation had a similar subscale mean score range (2.85–3.50), as did children with no fragile X (3.33–3.86). For five out of the eight subscales, children with the full mutation had the lowest scores indicating more sensory challenges; this was followed by children with an *FMR1* premutation who had the second lowest scores and then children with no fragile X who had the highest scores and thus fewer sensory issues. For the other three subscales – Affiliative touch aversion, Selective eating and Digestive problems – children with an *FMR1* premutation reported the highest level of sensory sensitivities followed by children with the full mutation and then children with no fragile X. The lowest rated subscales for children with an *FMR1* premutation, indicating the highest levels of sensory challenges, were Selective eating (2.95), Digestive problems (3.03), and Tactile hypersensitivity (3.11). Children with the full mutation had similar types and levels of sensory sensitivities with Auditory threat hypersensitivity (2.85) being the most challenging, followed by Selective eating (3.00), and Tactile hypersensitivity (3.01). Children with no fragile X had some challenges with selective eating (3.32) but had few sensory sensitivities on the other subscales. Median scores and interquartile ranges for the subscales are also reported and reflect the skewed nature of the data. However, median scores mimicked the ordering found for the mean scores, with children with no fragile X having the highest median scores, followed by children with an *FMR1* premutation, and then children with the full mutation. The exceptions to this were for the Selective eating and Digestive problems subscales which were lowest for children with an *FMR1* premutation.

**Table 2:** Mean subscale and item-level scores on the BBCSS.

	Typically developing (*n* = 33) Mean (*SD*)	*FMR1* premutation (*n* = 14) Mean (*SD*)	Fragile X syndrome (*n* = 129) Mean (*SD*)
Auditory threat hypersensitivity	3.56 (0.54)	3.32 (0.78)	2.85 (0.67)
Subscale median and interquartile range	3.7 (3.4–4.0)	3.4 (3.1–4.0)	2.9 (2.3–3.3)
Negative response to loud noise	3.46 (0.93)	3.18 (1.17)	2.71 (1.06)
Distracted by noise	3.39 (0.84)	3.00 (1.13)	2.02 (0.86)
Hands over ears	3.48 (0.81)	3.55 (0.82)	2.94 (1.00)
Trouble working with background noise	3.43 (0.74)	3.33 (0.89)	2.83 (0.98)
Upset by others’ cry	3.86 (0.47)	3.36 (1.03)	3.07 (0.92)
Distracted by continuous noise	3.54 (0.76)	3.18 (0.87)	2.93 (0.96)
Startles easily	3.70 (0.82)	3.36 (1.21)	3.00 (1.00)
Distracted by sound not heard by others	3.83 (0.39)	3.18 (0.87)	3.07 (0.96)
Negative reaction to places with noise	3.81 (0.60)	3.36 (0.92)	2.99 (1.03)
Auditory hyposensitivity to voices	3.59 (0.53)	3.53 (0.47)	3.21 (0.60)
Subscale median and interquartile range	3.8 (3.4–4.0)	3.6 (3.0–4.0)	3.3 (2.8–3.7)
Does not hear you	3.32 (0.77)	3.25 (0.87)	2.85 (0.82)
Does not respond to name	3.68 (0.55)	3.55 (0.52)	3.20 (0.75)
Speak loudly to get attention	3.70 (0.61)	3.82 (0.40)	3.50 (0.66)
Unaware of continuous noise	3.56 (0.71)	3.45 (0.69)	3.36 (0.80)
Long time to respond	3.77 (0.43)	3.50 (0.52)	3.14 (0.85)
Visual hypersensitivity	3.67 (0.58)	3.59 (0.45)	3.37 (0.60)
Subscale median and interquartile range	4.0 (3.6–4.0)	3.7 (3.3–4.0)	3.6 (3.0–3.8)
Bothered by bright lights	3.71 (0.72)	3.50 (0.53)	3.51 (0.68)
Covers eyes or squints	3.50 (0.86)	3.45 (0.69)	3.50 (0.72)
Unable to tolerate bright lights	3.67 (0.73)	3.40 (0.70)	3.54 (0.67)
Unable to tolerate flashing lights	3.65 (0.81)	3.56 (0.53)	3.48 (0.79)
Upset when exposed to bright lights	3.71 (0.78)	3.89 (0.33)	3.62 (0.73)
Sensitive to bright lights	3.50 (0.83)	3.60 (0.52)	3.64 (0.64)
Sensitive to flashing lights	3.67 (0.84)	3.78 (0.44)	3.63 (0.67)
Hesitates to go outside when sunny	3.89 (0.32)	3.78 (0.44)	3.73 (0.61)
Distracted by movement	3.70 (0.57)	3.80 (0.63)	2.92 (1.04)
Distracted by movement of objects	3.63 (0.60)	3.45 (0.82)	2.70 (1.02)
Tactile hypersensitivity	3.72 (0.46)	3.11 (0.79)	3.01 (0.70)
Subscale median and interquartile range	3.9 (3.6–4.0)	3.1 (2.5–3.8)	3.0 (2.5–3.6)
Distressed by tooth brushing	3.63 (0.89)	3.00 (1.32)	2.68 (1.15)
Distressed by face washing	3.65 (0.86)	3.22 (1.30)	2.77 (1.12)
Distressed by fingernail cutting	3.71 (0.59)	3.00 (1.50)	2.68 (1.12)
Distressed by hair brushing	3.63 (0.83)	2.70 (1.49)	2.98 (1.20)
Insists labels be removed from clothes	3.65 (0.67)	3.22 (0.83)	2.76 (1.22)
Refuses to wear certain fabrics	3.75 (0.55)	3.50 (0.76)	2.99 (1.07)
Complains clothes are tight or scratchy	3.60 (0.60)	3.33 (0.71)	3.13 (0.95)
Prefers not to wear clothes	3.82 (0.53)	3.71 (0.49)	3.38 (0.99)
Reaction to cold objects on hands	3.94 (0.25)	3.71 (0.49)	3.57 (0.72)
Reaction to cold objects on face	3.88 (0.34)	3.63 (0.52)	3.44 (0.82)
Affiliative touch aversion	3.79 (0.35)	3.37 (0.82)	3.40 (0.70)
Subscale median and interquartile range	4.0 (3.7–4.0)	3.8 (3.0–4.0)	3.7 (3.0–4.0)
Resists hugging	3.70 (0.47)	3.22 (1.09)	3.24 (0.96)
Negative reaction to hand holding	3.79 (0.42)	3.50 (0.76)	3.35 (0.94)
Reaction to being touched	3.94 (0.24)	3.22 (1.09)	3.57 (0.64)
Selective eating	3.32 (0.74)	2.95 (1.03)	3.00 (0.77)
Subscale median and interquartile range	3.5 (3.0–4.0)	3.0 (2.1–4.0)	3.2 (2.5–3.7)
Avoids certain tastes	3.04 (1.06)	2.75 (1.29)	2.60 (1.12)
Resists certain textures of food	3.17 (1.07)	2.45 (1.44)	2.47 (1.11)
Avoids certain food smells	3.12 (0.95)	2.91 (1.22)	2.93 (1.10)
Resists certain temperatures of food	3.71 (0.78)	2.91 (1.30)	3.04 (1.10)
Sucks on objects other than food	3.60 (0.91)	3.50 (0.76)	3.47 (0.88)
Eats less than appropriate	3.76 (0.56)	3.45 (0.82)	3.73 (0.65)
Ingestive problems	3.86 (0.34)	3.67 (0.50)	3.50 (0.65)
Subscale median and interquartile range	4.0 (4.0–4.0)	4.0 (3.7–4.0)	3.7 (3.0–4.0)
Gags often	3.84 (0.37)	3.56 (0.53)	3.20 (0.99)
Vomits often	3.94 (0.25)	3.88 (0.35)	3.50 (0.81)
Has difficulty swallowing solids	4.00 (0.00)	3.75 (0.71)	3.82 (0.51)
Digestive problems	3.74 (0.39)	3.03 (0.80)	3.46 (0.71)
Subscale median and interquartile range	4.0 (3.6–4.0)	3.0 (2.5–3.8)	3.8 (3.0–4.0)
Has acid reflux	3.88 (0.34)	3.13 (1.13)	3.44 (0.99)
Has excessive intestinal pain	3.88 (0.33)	3.00 (1.12)	3.48 (0.91)
Often constipated	3.68 (0.48)	3.10 (0.99)	3.49 (0.83)
Has stomach cramping	3.77 (0.43)	3.00 (0.82)	3.51 (0.80)

*Scores range from 1 to 4, low scores represent more problems, high scores represent optimal functioning. IQR, interquartile range.*

A similar pattern was found for many of the item-level scores, with children with the full mutation having the most sensory sensitivities and those with an *FMR1* premutation having similar but slightly fewer issues, followed by children with no fragile X with the fewest challenges. For children with the premutation, the lowest rated items, indicating more challenging sensory behaviors, were: Resists certain textures of food (2.45), distressed by hair brushing (2.70), avoids certain tastes (2.75), avoids certain food smells (2.91), and resists certain temperatures of food (2.91). Two of these items [resists certain textures of food (2.47), avoids certain tastes (2.60)] were among the lowest rated items for children with the full mutation and three [avoids certain tastes (3.04), avoids certain food smells (3.12), resists certain textures of food (3.17)] for children with no fragile X. The highest rated items, or those behaviors for which parents of children with the premutation indicated few sensory challenges, were: Upset when exposed to bright lights (3.89), vomits often (3.88), speak loudly to get attention (3.82), and distracted by movement (3.80). Only one of these items was among the highest rated for children with the full mutation [upset when exposed to bright lights (3.64)] and one for children with no fragile X [vomits often (3.94)].

### Differences in Sensory Behaviors by Child and Family Variables

We were also interested in examining whether there were differences in sensory behaviors by a variety of child and family characteristics. **Table 3** displays the group comparisons for child gender, age, autism status, and family income for each of the eight BBCSS subscales. Males had lower mean scores, and thus more sensory sensitivities, for the Auditory threat hypersensitivity (2.90), Auditory hyposensitivity (3.24), Tactile hypersensitivity (2.99), Selective eating (2.95), and Ingestive problems (3.47) subscales when compared with females (3.29, 3.45, 3.40, 3.31, and 3.82, respectively). Children who were co-diagnosed with autism exhibited more sensory sensitivities than those with FXS only for 5 of the 8 BBC subscales: Auditory threat hypersensitivity (2.75 vs. 3.15), Auditory hyposensitivity (3.04 vs. 3.43), Visual hypersensitivity (3.32 vs. 3.48), Tactile hypersensitivity (2.79 vs. 3.27), Affiliative touch aversion (3.25 vs. 3.56). Family income was shown to be statistically significant for two subscales. Children who were from lower income families (under $75,000) had higher mean levels of Auditory threat hypersensitivities (2.80) and Auditory hyposensitivities (3.18) when compared with children from higher income families (3.15 and 3.39, respectively). There were no differences across the BBCSS subscales when comparing children 12 years and under with those over 12 years of age.

**Table 3:** Non-parametric group comparison by child gender, age, autism status, and family income.

	Gender χ^2^ (*p*-value)	Age χ^2^ (*p*-value)	Autism status χ^2^ (*p*-value)	Income χ^2^ (*p*-value)
Auditory threat hypersensitivity	**12.14 (<0.001)**	0.68 (0.409)	**14.21 (<0.001)**	**9.37 (0.002)**
Auditory hyposensitivity	**5.77 (0.016)**	0.65 (0.421)	**16.16 (<0.001)**	**7.09 (0.008)**
Visual hypersensitivity	2.33 (0.127)	0.30 (0.585)	**4.82 (0.03)**	2.84 (0.092)
Tactile hypersensitivity	**12.77 (<0.001)**	0.65 (0.422)	**16.09 (<0.001)**	1.85 (0.174)
Affiliative touch aversion	2.30 (0.129)	0.78 (0.378)	**9.13 (0.003)**	0.10 (0.747)
Selective eating	**7.60 (0.006)**	0.19 (0.661)	3.23 (0.072)	0.22 (0.639)
Ingestive problems	**10.95 (<0.001)**	0.79 (0.375)	1.51 (0.219)	0.85 (0.356)
Digestive problems	0.28 (0.595)	0.81 (0.366)	1.20 (0.274)	0.01 (0.919)

*Omnibus Kruskal–Wallis test at p < 0.05. Bolded cells reached statistical significance.*

**Table 4** presents the results of a Kruskal–Wallis omnibus and *post hoc* comparison tests using a Bonferroni correction by fragile X status. The overall group test showed statistically significant differences in mean rank sum on 7 out of the 8 BBCSS subscales; Selective eating was the only subscale that was statistically equivalent for children with the premutation, children with the full mutation, and typically developing children. In *post hoc* comparisons between children with the full mutation and children with the premutation, there were no differences across the subscale. Thus, children with an *FMR1* premutation exhibited statistically the same number of sensory challenges as children with the full mutation. When comparing children with the premutation to those with no fragile X, we found one statistically significant difference on the Digestive problems subscale. Children with an *FMR1* premutation had more digestive problems than the typically developing controls. Finally, comparisons between children with the full mutation and those without fragile X showed differences on six of the eight subscales, including Auditory threat hypersensitivity, Auditory hyposensitivity, Visual hypersensitivity, Tactile hypersensitivity, Affiliative touch aversion, and Ingestive problems. Selective eating and Digestive problems were similar for children with the full mutation and no fragile X.

**Table 4:** Non-parametric group comparisons by fragile X status with *post hoc* analyses using Bonferroni correction.

	Omnibus group comparison χ^2^ (*p*-value)	Full mutation vs. Premutation χ^2^ (*p*-value)	Premutation vs. No fragile X χ^2^ (*p*-value)	Full mutation vs. No fragile X χ^2^ (*p*-value)
Auditory threat hypersensitivity	**31.69 (<0.001)**	5.56 (0.018)	0.40 (0.530)	**29.09 (<0.001)**
Auditory hyposensitivity	**13.26 (0.001)**	3.05 (0.081)	0.16 (0.692)	**11.47 (<0.001)**
Visual hypersensitivity	**11.06 (0.004)**	1.39 (0.239)	0.61 (0.433)	**10.48 (0.001)**
Tactile hypersensitivity	**21.78 (<0.001)**	0.27 (0.606)	5.53 (0.019)	**21.94 (<0.001)**
Affiliative touch aversion	**6.13 (0.047)**	0.02 (0.878)	1.70 (0.193)	**6.21 (0.013)**
Selective eating	4.70 (0.095)	0.01 (0.903)	0.85 (0.360)	4.86 (0.028)
Ingestive problems	**7.38 (0.025)**	0.42 (0.518)	2.32 (0.128)	**7.12 (0.008)**
Digestive problems	**7.37 (0.025)**	3.65 (0.056)	**7.55 (0.006)**	2.69 (0.101)

*Omnibus Kruskal–Wallis test at p < 0.05, post hoc Kruskal–Wallis comparisons with Bonferroni correction at p < 0.017. Bolded cells reached statistical significance.*

## Discussion

The goal of this study was to describe sensory processing in children with an *FMR1* premutation and to examine differences in sensory challenges when compared with children with the full mutation and those with no fragile X. Our findings add to the mounting evidence base that suggests that some individuals with an *FMR1* premutation have a similar, though less severe, clinical phenotype as those with FXS. These results, in particular, provide additional information about sensory challenges for a broad age range of both male and female children with an *FMR1* premutation, including those who were co-diagnosed with autism.

Across the majority (90%) of the BBCSS items, children with an *FMR1* premutation displayed more sensory challenges than typically developing children and also scored lower on about one-third (32%) of the items than children with the full mutation. This was especially true for digestive problems and tactile hypersensitivities which were among the highest rated sensory challenges for children with an *FMR1* premutation. Affiliative touch aversion was another area in which children with an *FMR1* premutation experienced challenges when compared to children without fragile X. Although children with an *FMR1* premutation also had issues with selective eating, these items were the lowest rated for typically developing children as well, thus reducing the difference between the two groups. When comparing item scores between the two fragile X groups, children with an *FMR1* premutation had similar types and levels of challenges as children with FXS. This was especially true for several items on the Visual hypersensitivity subscale. The exceptions to this were items on the Auditory threat hypersensitivity subscale which was more impaired in children with the full mutation and items on the Selective eating and Digestive problems subscales which was more impaired in children with an *FMR1* premutation.

Gender was related to many sensory challenges, with males typically having more sensitivities. Females, however, had more digestive problems. Autism status was also statistically significant in five of the eight subscales, with children who were co-diagnosed with autism having more sensory problems. These findings support earlier work which found similar patterns of hypersensitivities in young children with intellectual and developmental disabilities (although not specifically fragile X) and those with autism when compared with typically developing peers (Baranek et al., 2007). Moreover, other studies that have focused specifically on children with autism have consistently shown that high levels of hyporesponsiveness are associated with avoiding behaviors and social and communication impairments (Ben-Sasson et al., 2007; Boyd et al., 2010; Foss-Feig et al., 2012). Hypersensitivity in children with autism, on the other hand, has been often linked to sensory seeking behaviors and over focused attention (Liss et al., 2006). Children with autism have been reported to have more eating problems and more restrictive food interests than children without autism (Schreck et al., 2004; Cermak et al., 2010). Similarly, studies of children with FXS (Raspa et al., 2010) and other developmental delays (Williams et al., 2005) have found similar selective eating problems, often starting before 18 months. However, we did not find statistical differences on these BBCSS subscales by autism status.

When examining variation on the BBCSS subscale scores by fragile X status, differences were found between children with an *FMR1* premutation and those with no fragile X for digestive problems. Although not statistically significant given the Bonferroni correction, tactile hypersensitivities were fairly pronounced and likely didn’t meet the cut-off due to the small sample size. Notably, there were no statistical differences between children with an *FMR1* premutation and children with the full mutation; however, this also could be related to sample size. Children with the full mutation did, however, differ in their sensory sensitivities when compared with children with no fragile X.

The only earlier study of sensory challenges in children with an *FMR1* premutation found that infants and toddlers had high levels of hypo-responsivity and increasing levels of hyper-responsivity as they age, as measured on the Sensory Experiences Questionnaire (SEQ), when compared with typically developing controls (Wheeler et al., 2016). In a companion article to the current study that examined the psychometric properties of the BBCSS (Kolacz et al., 2018), we examined the relationship between the BBCSS subscales and the SEQ subscales and found moderate correlations between the Auditory threat hypersensitivity, Visual hypersensitivity, Tactile hypersensitivity, Affiliative touch aversion, and Selective eating subscales on the BBCSS and the SEQ’s Hyper-responsivity subscale. Data from the current study, which indicated more challenges for children with an *FMR1* premutation when compared to children with no fragile X on all four of these BBCSS subscales, therefore, are in keeping with the Wheeler and colleagues’ findings, and suggests a need for further research on the implications of hypersensitivities in the premutation. These elevations in hypersensitivities may be related to the increased risk for mood, anxiety, and autism features frequently reported in older individuals with a premutation (Bailey et al., 2008; Bourgeois et al., 2011). Despite moderate correlations in our companion article between the BBCSS Auditory hyposensitivity to voices subscale and the SEQ Hyposensitivity subscale, the current study did not find large hyposensitivity differences between children with an *FMR1* premutation and children with no fragile X, which could have been hypothesized based on the Wheeler and colleagues’ data. These results could reflect fewer auditory sensitivity issues in the premutation. It also could be a result of the older age of the current sample, as previous studies have suggested a hypo responsive pattern for infants and toddlers which appear to resolve or be overshadowed by increasing hyper sensitivities as they age.

These findings should be interpreted in the context of several limitations. First, although we had a large, geographically diverse sample of males and females with fragile X across a large age range, the majority of respondents were from White, upper-middle class households. This makes it difficult to extrapolate the findings to the broader fragile X population. Second, the sample of children with an *FMR1* premutation was small and may not be representative. However, in order to be conservative in our analytic approach, we used non-parametric tests to account for the small, skewed samples. Moreover, we did not make statistical comparisons between fragile X groups at the item-level due to the numerous tests to be run. Finally, all data are based on parent-report, including fragile X and autism status, and no clinical confirmation of these diagnoses was available. Although clinician report is often considered the gold standard, it is reasonable to assume that parents are accurate in their survey responses. In addition, the survey did not collect data from parents on CGG repeat number.

## Conclusion

These data provide further evidence that some children with an *FMR1* premutation experience sensory challenges that are similar to children with FXS but different than typically developing children. Data on sensory challenges and their interrelationship with other behavioral comorbidities, such as attention problems and anxiety, are needed in order to develop appropriate modifications and interventions. More broadly, research is needed to understand the natural history of those with an *FMR1* premutation. Both cross-sectional and longitudinal data across the lifespan will help to provide a better picture of how all individuals across the *FMR1* spectrum are affected, including which individuals are most at-risk for cognitive and behavioral challenges.

## Author Contributions

MR, ACW, JK, KH, and SP contributed to the conception and design of the study. AE coordinated data collection. AW organized the database and performed the statistical analysis. MR wrote the first draft of the manuscript. All authors contributed to manuscript revision, read and approved the submitted version.

## Conflict of Interest Statement

The authors declare that the research was conducted in the absence of any commercial or financial relationships that could be construed as a potential conflict of interest.
